# Distribution and Diversity of Bacteria and Fungi Colonization in Stone Monuments Analyzed by High-Throughput Sequencing

**DOI:** 10.1371/journal.pone.0163287

**Published:** 2016-09-22

**Authors:** Qiang Li, Bingjian Zhang, Zhang He, Xiaoru Yang

**Affiliations:** 1 Laboratory of Cultural Relics Conservation Materials, Department of Chemistry, Zhejiang University, Hangzhou, 310027, China; 2 Department of Cultural Heritage and Museology, Zhejiang University, Hangzhou, 310028, China; 3 Monitoring and Management Center of Hangzhou West Lake World Cultural Heritage, Hangzhou, Zhejiang, 310007, China; Free University of Bozen/Bolzano, ITALY

## Abstract

The historical and cultural heritage of Qingxing palace and Lingyin and Kaihua temple, located in Hangzhou of China, include a large number of exquisite Buddhist statues and ancient stone sculptures which date back to the Northern Song (960–1219 A.D.) and Qing dynasties (1636–1912 A.D.) and are considered to be some of the best examples of ancient stone sculpting techniques. They were added to the World Heritage List in 2011 because of their unique craftsmanship and importance to the study of ancient Chinese Buddhist culture. However, biodeterioration of the surface of the ancient Buddhist statues and white marble pillars not only severely impairs their aesthetic value but also alters their material structure and thermo-hygric properties. In this study, high-throughput sequencing was utilized to identify the microbial communities colonizing the stone monuments. The diversity and distribution of the microbial communities in six samples collected from three different environmental conditions with signs of deterioration were analyzed by means of bioinformatics software and diversity indices. In addition, the impact of environmental factors, including temperature, light intensity, air humidity, and the concentration of NO_2_ and SO_2_, on the microbial communities’ diversity and distribution was evaluated. The results indicate that the presence of predominantly phototrophic microorganisms was correlated with light and humidity, while nitrifying bacteria and *Thiobacillus* were associated with NO_2_ and SO_2_ from air pollution.

## Introduction

A large percentage of the world’s stone cultural heritage monuments have suffered severe and irreversible degradation and deterioration from microorganisms [1–4]. This damage and deterioration not only causes the loss of aesthetic value but also presents challenges to researchers exploring the evolution of ancient civilizations. In recent decades, a variety of epilithic and endolithic microbial communities have been reported in limestone from archaeological sites all over the world, revealing that these microbial communities have distinct diversity patterns under different environmental conditions. As a consequence, the microbial colonization and biodeterioration of building stones is usually linked to environmental conditions [5,6]. The most significant parameters affecting microbial colonization are physical factors, mainly humidity, air temperature, light intensity, and the composition of air pollutants, as well as the chemical nature, mechanical strength, solubility and porosity of the substratum, which are modified in polluted environments [7–10]. In general, microbial colonization is initiated by a wide variety of phototrophic microorganisms, mainly cyanobacteria and algae [11–13]. Colonization of stone monuments by predominately light-dependent phototrophic types, including Cyanobacteria, algae and lichens, gathers organic matter in the form of dead cells and trapped debris so that heterotrophic or chemoorganotrophic fungi and bacteria can grow on the surface of the stone [14]. Cyanobacteria are oxygenic and phototrophic bacteria and have been suggested to be of greater ecological importance than other organisms on the exposed stones surfaces of buildings, since they act as pioneer organisms and may have the most significant influence on the deterioration of exposed stone [15–18]. Their tolerance for water stress and variable light intensity helps explain their frequent occurrence on stone monuments even where the light intensity is low. Atmospheric pollutants, such as nitrogen oxides and sulfur dioxide, are also a dominant factor in accelerating damage to and deterioration of exposed stone artwork. These gases are oxidized to form nitrous acid, nitric acid and sulfuric acid, which are deposited onto the monuments’ surface and cause severe corrosion of the stone matrix. However, the oxidation and reduction reactions are also mediated and catalyzed by enzymes which are produced by *Thiobacillus* or Nitrobacteria inhabiting the surface of stone cultural artworks [19,20]. Nitrifying bacteria are chemolithotrophs which oxidize nitrogen or nitrogen dioxide for energy and transform them into nitric and nitrous acid. *Thiobacillus* have the ability to convert inorganic sulfur to sulfate in the form of sulfuric acid. Many reports have shown that nitrifying and sulfur-oxidizing bacteria are present on stone monuments [21,22]. Previous studies have shown that stone monuments in subterranean environments suffer corrosion primarily because of the phylum Actinobacteria; the genera *Streptomyces* and *Nocardia* form a large part of the population and play an important role in the colonization of the surface of stone murals [23,24]. However, enough attention has not been paid to colonization by Actinobacteria in outdoor environments.

**Fig 1:**
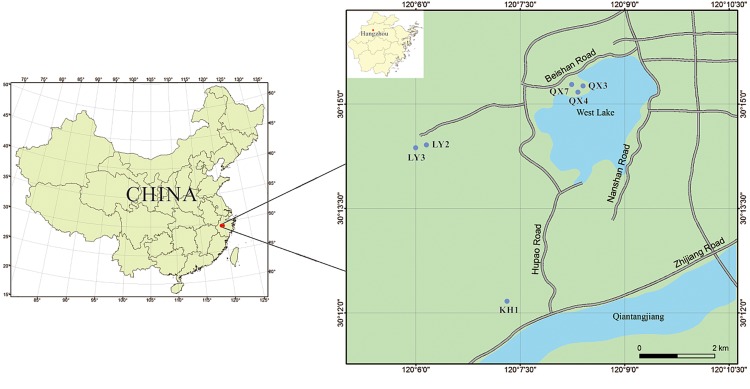
Sampling site locations.

The 3 sampling sites in Qingxing palace, Kaihua temple and Lingyin temple are in the West Lake Cultural Landscape site, which was added to the World Heritage List in 2011 for its graceful landscape and historical importance (Fig 1). These sites are distributed around West Lake in Hangzhou, China. The ancient monument in the Kaihua temple was built during the Northern Song dynasty (960–1219 A.D.) and was excavated in 2009. It is an important example of the Liuhe pagoda layout for “the left tower and right courtyard” in ancient China. The Qingxing palace monument is located south of the mountain Gu and was built during the Qing dynasty. The main body of the original buildings was destroyed, leaving only the overall layout of the site. The preserved artwork consists mainly of a stone pavilion, white marble pillars, the rockery and stone carvings. The Buddhist stone pillars of the Lingyin temple are distributed on all sides of the temple inlet and were built during the Five Dynasties period or early Northern Song dynasty of ancient China. They are recognized as the some of the most historical and valuable monuments of the Lingyin temple because of their exquisite Buddhist statues. The 3 sampling sites have a subtropical monsoon climate with an average annual temperature of 17.8°C, an average relative humidity of 70.7%, and an average yearly rainfall of 1454 mm. The temperature and high humidity and are suitable for the growth of photosynthetic microorganisms and enhance their destructive activity. However, the six sampling sites showed different microbial distributions and diversity depending on their environmental character.

The aims of our investigation were to identify the microflora colonizing the different sampling sites using high-throughput sequencing of 16S rDNA for bacteria and ITS sequences for fungi; to elucidate the diversity and distribution patterns of the microbial communities colonizing different stone monuments and explain the relationship between these communities and environmental factors, including light intensity, air humidity and concentration of NO_2_ and SO_2_; and to assess the ability of these microorganisms to degrade and damage stone monuments and suggest effective control measures.

## Materials and Methods

### Sample collection and description

Monitoring and Management Center of Hangzhou West Lake World Cultural Heritage issued the permission for each sample collection. The samples were collected from 3 locations distributed around West Lake, Hangzhou, and will be referred to as KH1, LY2, LY3, QX3, QX4 and QX7 (S1 Fig). They were taken from six different places with signs of microbial biofilms. Sample KH1 was collected from green biofilms present on the surface of ancient bricks located in Kaihua temple (S1 Fig). Samples LY2 and LY3 were collected from green and white colonies located on a Buddhist stone pillars of Lingyin temple (S1B and S1C Fig). Samples QX3 and QX4 were collected from brown and black colonies present on dragon carvings on a column pedestal made of white marble (S1D and S1E Fig), and QX7 was taken from green biofilms covering the surface of a stone building built during the Qing dynasty (S1F Fig). All samples were collected as intact and aseptically as possible. Samples were collected into sterile tubes and stored at -20°C for further analysis. A portion of each sample was taken for morphological and mineralogical analysis by scraping with a sterile scalpel. The remaining portion of each sample was used for high-throughput sequencing analysis. Sampling was carried out with the assistance of the West Lake World Cultural Heritage monitoring center.

### Environmental data acquisition

Long-term meteorological data were collected and monitored around the West Lake World Cultural Heritage sites. Temperature and relative humidity were measured with an aspiration psychrometer (Germany, 174H). Nitrogen dioxide and sulfur dioxide content were measured by a nitrogen dioxide detector (GDYK-501S) and a sulfur dioxide detector (GDYK-402S). Light intensity was measured with a photometer (DT-1309). For our analysis, we used the monthly average of the environmental parameters corresponding to the sampling time.

### SEM-EDS analysis

Morphological and mineralogical structure characterization was carried out with a scanning electron microscope (Hitachi S-3600N, Japan) that includes an Energy-Dispersive Spectroscopy (EDS) probe. Fragments of samples with microbial colonies were glued to a double-sided conductive carbon tab stuck on a standard vacuum-clean stub which was coated with gold for 90 seconds in vacuum conditions.

### DNA extraction and high-throughput sequencing of 16S rDNA and ITS

Total genomic DNA was extracted from the microbial samples using the Power Soil^®^ DNA Isolation Kit (MO BIO Laboratories, Inc., CA, USA) according to the manufacturer’s instructions. The V4 region of the 16S rRNA gene was amplified using the bacterial/archaeal 515F-806R primer set [25]. The primer sequences were: 515F (GTGCCAGCMGCCGCGGTAA) and 806R (GGACTACHVGGGTWTCTAAT). The ITS2 region of the ITS gene was amplified using the fungal ITS3-ITS4 primer set. The primer sequences were: ITS3 (GCATCGATGAAGAACGCAGC) and ITS4 (TCCTCCGCTTATTGATATGC). Illumina sequencing was performed on a MiSeq platform by the Beijing Genomics Institute (BGI). The general analysis process was as follows: Bar-coded fragments spanning the bacterial/archaeal/fungal hypervariable region were amplified from DNA and these amplicons were bidirectionally sequenced using the MiSeq platform. For each library, the forward and reverse sequences derived from the same DNA fragment were assembled using FLASH software (Fast Length Adjustment of Short reads, v1.2.11) [26]. Clean tags were spliced from paired end reads through the overlap relationship of different reads.

OTU cluster analysis was carried out with clean tags using the USEARCH software (v7.0.1090) [27]. The classification of the species was processed and analyzed by OTU annotation using the RDP Classifier software (v2.2). Using the UPARSE software, representative OTU sequences were generated using a 97% sequence-identity threshold. Chimeric sequences were identified and removed using the UCHIME software (v4.2.40) [28]. The 16S rDNA and ITS chimeric sequences databases used were gold database (v20110519) and UNITE (v20140703).

### Processing of sequencing data and statistical analysis

OTU rank curves and Principal Component Analysis (PCA) were generated using the R Package vegan. A heatmap was constructed using the R package gplots using R software (v3.1.1). A phylogenetic tree was constructed using FastTree with Kimura’s two-parameter model. Alpha diversity, including the observed species index, Chao1, ACE, Shannon, and Simpson indices, were analyzed using Mothur (v1.31.2) and R value. Bray–Curtis distances correspond to the frequency of sequences belonging to high-rank archaeal, bacterial, and eukaryotic taxa. Principal coordinates analysis (PCoA) was conducted on the diversity of different genera using QIIME software (v1.80) [29]. Raw sequences were deposited in the NCBI Sequence Read Archive (SRA) database (accession number: SAMN05559798- SAMN05559803).

## Results

### Sample description and SEM-EDS analysis

Six samples were collected from the West Lake Cultural Landscape, which is a World Cultural Heritage site and includes many historic sites, such as the Feilaifeng shike, Lingyin and Kaihua temple, Qingxing palace and Qiantang sites. In our study, we sampled from three sites located in different environmental conditions in order to analyze the richness and diversity of microbial communities. S1 Fig shows the diverse shapes and colors of the microbial colonizers present in the ancient stone and brick monuments which were preserved in different conditions. The green colonizers shown in S1A–S1F Fig are probably mainly composed of photosynthetic bacteria and algae; they were widely distributed on the surface of ancient artworks which exposed to sunshine and rainfall throughout the year. The white plaque, which appeared in the form of scattered points, was also commonly present on the cave statues, as shown in S1C Fig. Samples LY2 and LY3 were collected from Buddhist stone pillars and represent different colonizers. Samples QX3, QX4 and QX7, which were taken from a variety of cultural monuments located in Qingxing palace, also represent diverse microbial colonies. Samples QX3, QX4, QX7, LY2 and LY3 were taken from stone monuments, while KH1 was taken from brick artwork.

**Fig 2:**
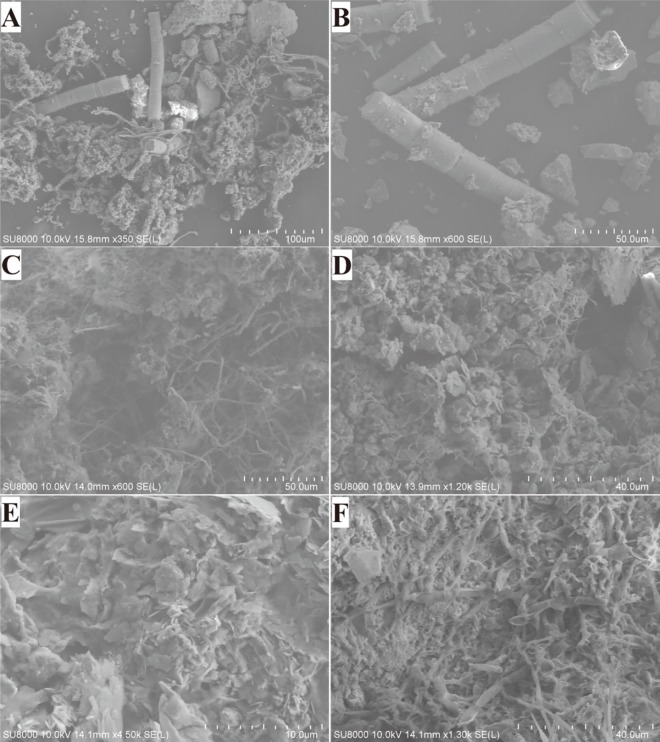
Scanning electron micrographs of microbial colonies from the stone monuments. A and B show algae and the fungal hyphae forming an aggregate with the archaeological monument’s matrix in sample KH1. The images presented in C and D show Actinobacteria filaments with a diameter of 0.5–2 μm and fungal filaments with a diameter of 2–4 μm in samples LY2 and LY3. E and F show lichens growing on the surface of the stone statues and forming a foliated structure in samples QX3 and QX7.

To investigate the microbial deterioration and colonization of the stone monuments, representative samples of the green biofilms, white plaque, and brown and black colonies on the surface of different sampling sites were analyzed using a scanning electron microscope and energy spectrum analysis. Fig 2 shows the microbial inhabitants colonizing the stone monuments and the extracellular polymeric substances (EPS) which they formed. Fig 2A and Fig 2B show algae and fungal hyphae forming an aggregate with an archaeological monument’s matrix. Fig 2C and Fig 2D show Actinobacteria filaments with a diameter of 0.5–2 μm and fungal filaments with a diameter of 2–4 μm. Fig 2E and 2F show lichens growing on the surface of stone statues and forming a foliated structure. Lichens are microbial in the sense that they consist of algal and fungal cells in close association, forming a visible thallus. Lichens not only cause damage to stone monuments through penetration of the substratum by fungal hyphae but also aggregate water and organic matter which can lead to other microbial growth.

The elemental composition of the stone monuments’ substrate is shown in S2 and S3 Figs. Based on the data shown in S2 Fig, we conclude that the composition of the white marble in Qingxing palace is aluminum oxide and silicon dioxide. Furthermore, iron elements were detected in sample QX4, according to the Energy Dispersive Spectrometer (EDS) analysis. We propose that the presence of iron will accelerate colonization by iron-oxidizing bacteria and discoloration and patina formation on the stones. The EDS analysis of stones collected from samples LY2 and LY3 shows that these stones mainly consist of sulfur, silicon, oxygen and calcium, with small amounts of carbon and aluminum (S3 Fig). These results reveal that the stones are comprised of a series of compounds including calcium silicate, aluminum oxide, silicon dioxide and sulfide. However, the presence of sulfur in the stones is probably associated with the SO_2_ detected in air.

### General analyses of microbial community diversity

High-throughput sequencing with an Illumina MiSeq platform was used to analyze the diversity and variability of the microbial communities colonizing the stone brick monuments. A total of 720,259 tags were optimized from the original reads. 1,928 bacterial and 490 fungal operational taxonomic units (OTUs) were obtained from the Qingxing palace samples, while 3,016 bacterial and 549 fungal OTUs were obtained from samples KH1, LY2 and LY3. In addition, a total of 530,424 tags were removed as chimeras. The range of bacterial OTUs from the six samples was 477 to 2,179, while the range for fungi was 213 to 416 (S1 and S2 Tables). The rarefaction curves of the bacteria and fungi tended to flatten, as shown in S4 Fig. OTU rank curves, which represent the degree of species richness and evenness, were constructed based on the OTUs of the six samples.

The alpha diversity was estimated based on the observed species, Chao1, ACE, Shannon and Simpson indices and reflected the community diversity of single samples (S1 Table). The observed species index, Chao1 index and ACE index reflect the species richness of sample communities, while the Shannon and Simpson index reflect species diversity. The observed species score of the bacterial communities ranged from 477 to 2,179, while the Chao1 and ACE scores ranged from 636 to 2,241 and from 621 to 2,268, respectively. All three indices indicated that LY2 had the highest and QX4 the lowest species richness. QX4 and QX7 had the most similar scores of all six samples. The Shannon and Simpson scores ranged from 2.23191 to 4.813508 and 0.04055 to 0.355860, respectively. LY2 had the highest score for observed species, Chao1 and ACE indices and the lowest score for the Simpson index, while the sample QX4 had the opposite pattern. These results demonstrate that the Qingxing palace samples had lower bacterial diversity and the Lingyin temple samples had the highest bacterial diversity.

The observed species score for the fungal communities ranged from 213 to 416, and the Chao1 and ACE scores from 146 to 436 and from 145 to 435, respectively. The Shannon and Simpson scores ranged from 1.239379 to 3.354797 and 0.074251 to 0.434495, respectively. LY3 had the highest fungal score for the observed species, Chao1, ACE and Shannon indices, and QX4 had the lowest score. These results indicate that the Lingyin temple samples had the highest fungal diversity (S2 Table).

The beta diversity index was used to analyze the difference in microbial community diversity between pairs of samples. The analysis was carried out using QIIME (v1.80), and the number of sequences from the six samples needed to be unified. The Bray-Curtis index was used to calculate and evaluate differences between each pair. S5 Fig shows a low bacterial diversity distance between samples LY2 and LY3. However, fungal diversity distance indicates that LY3 had a similar beta diversity index to QX3, indicating that these two samples had fungal communities with similar diversity.

### Microbial community diversity and richness analysis

**Fig 3:**
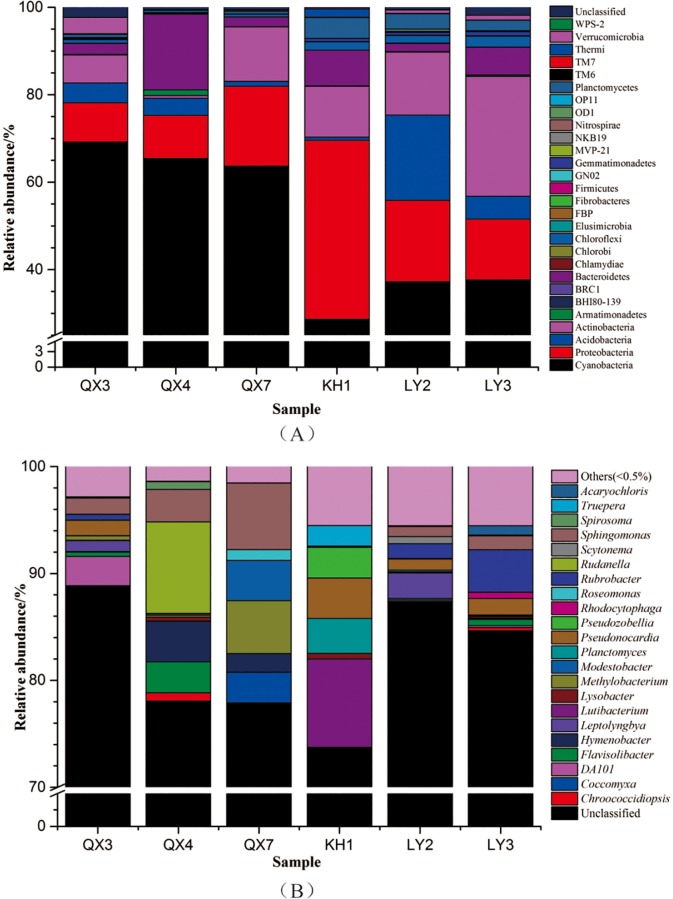
Distribution patterns of bacterial phyla (A) and genera (B) in the six samples.

Metagenomic sequencing of the bacterial 16S rDNA and fungal ITS sequences was used to analyze the diversity and community richness of the six samples. The distribution of different phyla in the samples is summarized in Fig 3A. The following 28 different phyla or groups make up most of the bacterial communities (with the remainder consisting of unclassified bacteria): Cyanobacteria, Proteobacteria, Acidobacteria, Actinobacteria, Armatimonadetes, BHI80-139, BRC1, Bacteroidetes, Chlamydiae, Chlorobi, Chloroflexi, Elusimicrobia, FBP, Fibrobactteres, Firmicutes, GN02, Gemmatimonadetes, MVP-21, NKB19, Nitrospirae, OD1, OP11, Planctomycetes, TM6, TM7, Thermi, Verrucomicrobia, WPS-2. Cyanobacteria were detected in all six samples and are the primary component of the bacterial communities, accounting for an average 50.31% of all bacterial phyla. Cyanobacteria accounted for the largest proportion of the communities collected from Qingzing palace, QX3 (69.22%), QX4 (65.47%) and QX7 (63.68%). The phyla Proteobacteria and Actinobacteria are the second and third largest members of the bacterial communities except in LY2, accounting for an average 18.41% and 12.17% of the communities, respectively. Proteobacteria were detected in all six samples and accounted for 40.92% of all bacterial phyla in KH1. Actinobacteria were detected in all six samples, with the largest proportion being 27.43% in sample LY3. In LY2, Acidobacteria was the second largest bacterial phylum, accounting for 19.48% of the community.

The relative abundance and distribution of bacterial genera in the samples is shown in Fig 3B. The bacterial communities consisted of *Acaryochloris*, *Chroococcidiopsis*, *Coccomyxa*, *Flavisolibacter*, *Hymenobacter*, *Leptolyngbya*, *Lutibacterium*, *Lysobacter*, *Methylobacterium*, *Modestobacter*, *Planctomyces*, *Pseudonocardia*, *Pseudozobellia*, *Rhodocytophaga*, *Roseomonas*, *Rubrobacter*, *Rudanella*, *Scytonema*, *Sphingomonas*, *Spirosoma*, *Truepera*, DA101, Unclassified, and Others (<0.5%). Unclassified genera were dominant in all six communities and accounted for an average of 81.74% of all bacterial communities. The genus *Lutibacterium* was the single dominant classified genus in KH1, followed by *Pseudonocardia*, *Planctomyces*, *Pseudozobellia* and *Truepera*, which accounted for 3.79%, 3.26%, 2.87% and 1.92% of the community, respectively. In LY2, the predominant identified genera were *Lutibacterium*, *Rubrobacter* and *Pseudonocardia*, accounting for 2.38%, 1.37% and 1.05% of the community. The genera *Rubrobacter*, *Pseudonocardia* and *Spirosoma* dominated the bacterial community in LY3, representing 3.98%, 1.55% and 1.29% of the total, respectively. *Pseudonocardia* and *Sphingomonas* appear in QX3, where they make up 1.45% and 1.52% of the community, respectively. *Rudanella* is the largest member of QX4, accounting for 8.55% of all classified genera, followed by the genera *Hymenobacter*, *Sphingomonas* and *Flavisolibacter*, which make up 3.80%, 3.03% and 2.89% of the community, respectively. The bacterial composition of QX7 was less diverse than that of the other samples, but great variation was observed in bacterial abundance. *Sphingomonas* accounted for the largest proportion, with a value 6.20%. *Methylobacterium*, *Modestobacter*, *Coccomyxa*, *Hymenobacter* and *Roseomonas* accounted for 4.96%, 3.76%, 2.85%, 1.73% and 1.02% of the community, respectively.

**Fig 4:**
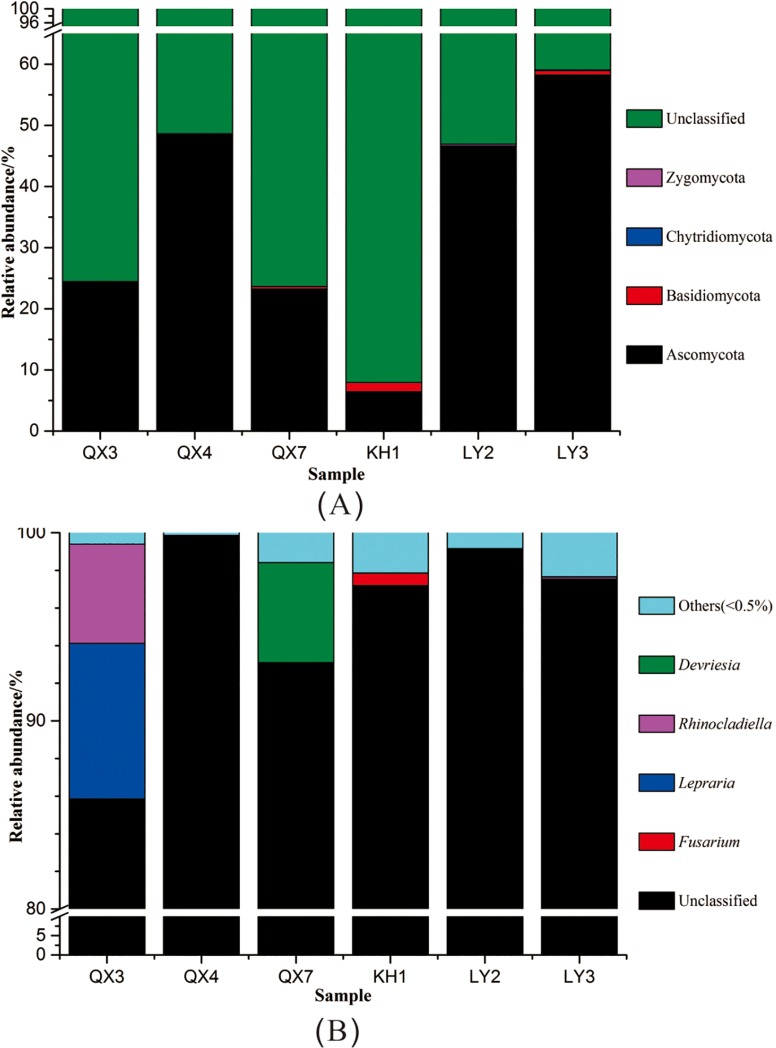
Distribution patterns of fungal phyla (A) and genera (B) in the six samples.

The fungal composition of all six samples was less diverse than that of the bacterial communities (Fig 4A and 4B). Unclassified fungi made up most of the communities, accounting for an average of 64.89% in all samples. The predominant classified fungal phyla were Ascomycota, Basidiomycota, Chytridiomycota and Zygomycota, accounting for an average of 34.56%, 0.47%, 0.03% and 0.05%, respectively. Ascomycota appeared in all of six samples and was the largest identified fungal community, making up 6.41%, 58.23%, 23.25%, 46.48%, 24.37 and 48.62% of KH1, LY3, QX7, LY2, QX3 and QX4, respectively. The fungal genera *Devriesia*, *Fusarium*, *Lepraria* and *Rhinocladiella* were present in all six samples but only accounted for an average of 0.12%-1.38%. *Lepraria* was the largest genus in QX3, where it accounted for 8.25%, followed by *Rhinocladiella*, which made up 5.28% of the community. *Devriesia* was the dominant classified genus in QX7, accounting for 5.31% of the community.

### The influence of environmental factors on the microbial distribution

Environmental factors, including temperature, light intensity, NO_2_ and SO_2_ concentration, and the relative humidity of the atmosphere and the monument’s surface, were assembled and evaluated using statistical analysis methods. The results are shown in S6 Fig. The temperature of Qingxing palace sampling sites was lower than those in the Kaihua temple and Lingyin temple. The lower temperature is less suitable for the growth of microbial communities, leading to decreased biodiversity. However, the light intensity of sites QX3, QX4, and QX7 was greater than at the other 3 sites. Since microbial colonization, particularly outdoors, is generally initiated by a variety of predominantly phototropic microorganisms, such as cyanobacteria, algae and lichens, followed by other heterotrophic microorganisms, higher light levels will accelerate colonization by phototropic microorganisms, leading to irreversible deterioration and damage to ancient stone and brick artwork. In addition, higher relative humidity would promote colonization by phototropic microorganisms (Fig 3A and 3B). In Fig 3A, the phylum Cyanobacteria accounted for almost twice as much of the community in QX3, QX4 and QX7 as in the other three samples. The average NO_2_ and SO_2_ concentrations were highest at the Lingyin temple, where they were 0.04 mg/L and 0.02 mg/L respectively, followed by the Qingxing palace, which had concentrations of 0.03 mg/L and 0.01 mg/L. The presence of NO_2_ and SO_2_ promotes the aggregation of nitrobacteria and sulfide-oxidizing bacteria which accelerate corrosion of the stones and bricks. The phylum Nitrospirae was present in sample KH1, LY2, LY3, QX3 and QX7, accounting for 0.008%, 0.22%, 0.11%, 0.02% and 0.004% of the communities, respectively. However, no nitrobacteria were detected in QX4. The Nitrospirae distribution is in accordance with the NO_2_ and SO_2_ concentrations.

## Discussion

Microorganisms play a crucial role in mineral transformation in the natural environment, notably in the formation of soils from rocks and the cycling of elements such as nitrogen and sulfur [30]. However, these processes cause irreversible damage to ancient stone monuments of cultural and historical significance. Although many reports have focused on the diversity and distribution of the microbial communities present on ancient stone monuments, no consistent conclusion has been reached [31–34]. Some workers consider lichens and fungi to be the primary factors leading to decay, while others have suggested that biological attack might be followed by physical and chemical agents. The microflora on external stone surfaces represent a complex ecosystem including not only bacteria, fungi, algae and lichens but also protozoa. Although collected from sites with similar signs of biodeterioration and microbial colonization, the microbial communities in this study varied enormously in composition because of differing environmental conditions and detection measures. In general, culture-independent measures are considered more convenient and informative than culture-dependent methods, which only enable the detection of 1–5% of the total microbial community [35]. In our study, a metagenomic approach was used in order to better understand the microbial biodiversity present in each sample. Metagenomics represents an indispensable tool since it is now accepted that, in most cases, the behavior of species can only be explained by taking into account the entire microbial community. Metagenomic methods have been gaining popularity for the detection and analysis of microbial communities on stone archaeological monuments.

The high-throughput sequencing results in our study revealed a rich diversity of bacterial communities on the stone archaeological samples. We identified 17 annotated bacterial phyla and 21 bacterial genera in the samples. Predominant among these was the bacterial phylum Cyanobacteria, which consists of phototrophic bacteria that can colonize rocks and stone monuments and produce aesthetic changes due to stains, colored biofilms and incrustations [36–38]. They are also considered pioneer colonizers along with other autotrophic microorganisms. Cyanobacteria were present in all six samples and accounted for approximately 50.3% of all bacteria communities. The Cyanobactiera genera *Chroococcidiopsis*, *Coccomyxa* and *Scytonema* were detected in our samples and are also frequently present in many stone monuments all over the world. Multiple reports show that Cyanobacteria have been identified in many stone monuments at sites with high humidity and illumination and high porosity of stone matrix fissures [39, 40]. In our study, the light intensity and the humidity of the air and the monuments were measured using professional testing instruments at all six sampling sites. Our results indicate that the light intensity of the Qingxing palace site was the greatest, followed by Lingyin and Kaihua temple. The relative air humidity at all six sampling sites followed a similar pattern, with the highest air humidity at Qingxing palace and lower humidity at Lingyin and Kaihua temple. Combined with the high-throughput sequencing results, we conclude that colonization by Cyanobacteria was correlated with the air humidity and light intensity at the six sampling sites. From Fig 3A, we can see that Cyanobacteria are the dominant members of QX3, QX4 and QX7 and account for 69.2%, 65.5% and 63.7% of all bacterial communities in those samples. However, the proportion of Cyanobacteria in LY2, LY3 and KH1 was only 37.2%, 37.6% and 28.6%, respectively. Furthermore, Cyanobacteria are probably the most resistant of the microbial communities on the surface of stone monuments, given their ability to resist high radiation and dehydration. Their ability to resist high UV radiation and dehydration conditions makes them among the most frequent microbial types on stone monuments [41].

Proteobacteria, Firmicutes, Acidobacteria and Actinobacteria are also frequently present on ancient mural paintings [42–44]. Proteobacteria and Firmicutes are associated with earthy and arid environments, while Actinobacteria are considered the dominant microbial taxon in subterranean environments, such as mural paintings in caves and on tombs [2]. In our study, Actinobacteria, represented by *Pseudonocardia* and *Rubrobacter*, accounted for 6.4%, 0.6%, 12.5%, 11.6%, 14.4% and 27.4% of the communities in QX3, QX4, QX7, KH1, LY2 and LY3, respectively. These results show that Actinobacteria were not only the predominant microorganisms in subterranean environments but also in outdoor environments exposed to light. Actinomycetes are important endoliths in all kinds of stone monuments, and many reports have emphasized their ability to deteriorate and degrade ancient archaeological monuments [45]. Chemolithotrophic microorganisms, such as sulfur oxidizers and nitrifying bacteria, also play a significant role in the biodeterioration and degradation of stone monuments [18]. These bacteria can convert inorganic sulfur or nitrogen compounds into inorganic acids, including sulfuric acid and nitric or nitrous acid, causing severe irreversible damage to stone monuments. In our study, the phylum Nitrospirae was present in KH1, LY2, LY3, QX3 and QX7, accounting for 0.008%, 0.22%, 0.11%, 0.02% and 0.004% of the communities, respectively. The highest average concentrations of NO_2_ and SO_2_ were at the Lingyin temple site, which had 0.04 mg/L and 0.02 mg/L respectively, followed by the Qingxing palace sample, with 0.03 mg/L and 0.01 mg/L. The Nitrospirae distribution is consistent with the concentration of NO_2_ and SO_2_, leading us to conclude that air pollutants such as NO_2_ and SO_2_ can accelerate growth of microbial inhabitants. S6 and S3 Figs show that a high concentration of sulfur was present in samples LY2 and LY3, with lower levels found in the other samples. We conclude that some sulfur-oxidizing bacteria, making up less than 0.01% of the community, converted SO_2_ pollutants in the air into sulfuric acid and sulfurous acid though oxidation and reduction reactions induced by biological enzymes.

The influence of fungi on the stone monuments is due to physical and chemical actions which are often associated with microbial deterioration and degradation. In general, fungal filaments, or mycelia, can assemble together and penetrate underlying substrates, as well as secreting enzymes and other extracellular products, such as organic acid and colored matter. In our study, there was low biodiversity of fungi at the level of phyla and genera compared with bacterial communities. Ascomycota was the predominant fungal taxon with an average of 34.56% of all six samples. It has long been known that fungi isolated from soils and weathering rock can solubilize a range of synthetic and natural silicates by producing various organic acids [46].

**Fig 5:**
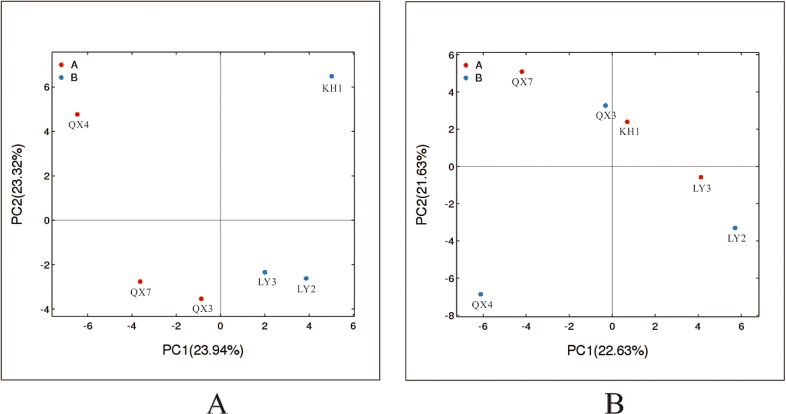
Principal component analyses of the (A) bacterial and (B) fungal communities in the six samples.

The structure of the microbial communities differed among the six samples, and the distribution patterns of the various microorganisms were irregular, along with differences in environmental conditions and stone substrate composition. The diversity and distribution of the microbial communities was assembled and evaluated using statistical analysis methods; the results of the principal component analysis (PCA) show that the bacterial structures of the communities in LY2 and LY3, QX3 and QX7 are similar (Fig 5A). However, samples KH1 and QX4 showed more diversity than the other samples. On the other hand, the fungal community structures differed greatly, according to the principal component analysis results (Fig 5B).

**Fig 6:**
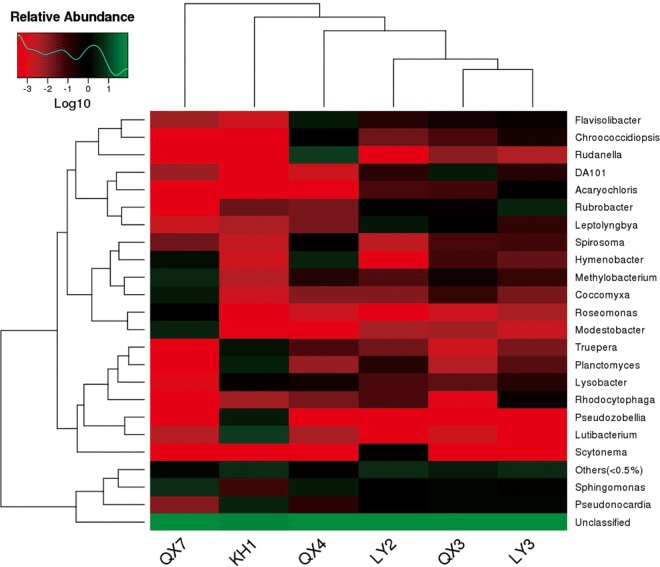
16S rRNA sequencing reveals the relationship and membership of the colonies from the stone monuments. Weighted UniFrac UPGMA tree based on bacterial and archaeal V4 16S rRNA gene sequences obtained from the six stone monuments located in Hangzhou city, Zhejiang province. The heat map shows the relative abundance within each sample of the 24 bacterial classes that were most abundant in the entire dataset. The abundance data were normalized by range-scaling each class Log_10_ (-3–1).

Among the classified bacterial taxa, *Pseudonocardia* and *Sphingomonas* seem to act as major players in the deterioration of stone monuments because of their high relative abundance and frequency (Fig 6). Species of *Pseudonocardia* were present in nearly all of the six samples and showed extremely high abundance in samples LY2 and LY3. *Sphingomonas* showed high relative abundance in all samples except for KH1. Among the classified fungal communities, *Lepraria* and *Rhinocladiella* were abundant in sample QX3, while *Devriesia* was abundant in QX7 (S7 Fig). Unclassified bacterial genera were dominant in all six communities, accounted for 88.7%, 78.1%, 77.9%, 73.7%, 87.3% and 84.6% of the communities in QX3, QX4, QX7, KH1, LY2 and LY3, respectively. However, unclassified bacterial phylum were present in all six samples but only accounted for an average of 1%. These results show that more bacterial communities can be identified to phylum rather than the genus. Unclassified fungus genera were also dominant in all six communities, accounted for 85.9%, 99.9%, 93.1%, 97.2%, 99.2% and 97.5% of the communities in QX3, QX4, QX7, KH1, LY2 and LY3, respectively. Unclassified fungus phylum accounted for an average of 64.7%. These results show that most of the microorganisms were present in all six samples belonged to unclassified microbial communities.

In conclusion, the abundant microbial colonization of three archaeological sites will cause irreversible damage to stone monuments. Sulfur cycle bacteria can convert limestone into gypsum, this can lead to the formation of dark surface colorations. Dark and green filamentous Cyanobacteria or fungus can form thick crusts on the surface of stone monuments. Apart from their aesthetic effects, these crusts can also result in water retention and subsequent spalling of stone [9]. Nitric and nitrous acids produced by some microorganisms may dissolve stone matrix and accelerate stone degradation. In our study, we show a positive association between the diversity and distribution of microbial communities and environmental parameters, including air humidity, illumination intensity, and the concentration of NO_2_ and SO_2_. As a consequence, the removal of the microbial colonies from the surface of stone monuments is an intervention that must be carefully evaluated. We must take into account all the interactions, including those between microbial communities and environmental factors. For instance, the removal of the initial microbial colonizers may trigger a succession of new microbial inhabitants which may cause more severe damage than the old microbial populations; furthermore, the inhibition of specific groups may favor the growth of others. The methods used to control the biodeterioration of stone monuments must therefore be interdisciplinary, drawing on microbiology, chemistry, physics, geology and ecology. In recent decades, synthetic materials such as epoxy, acrylic resins and polyurethane have been widely used to protect and consolidate stone monuments. However, the biodeterioration of these polymers by microbial communities should be considered and long-term detection must be performed. Furthermore, environmental factors should be maintained within a range suitable for the stone monuments while providing protection against colonization by microorganisms.

## Supporting Information

S1 Fig The stone monuments are heavily colonized by green and white biofilms. (A) shows sample KH1, which was collected from the green biofilms on the surface of the ancient bricks located in Kaihua temple. (B) and (C) show samples LY2 and LY3, which were collected from green and white colonies located on the Buddhist stone pillars of Lingyin temple. (D) and (E) show samples QX3 and QX4, which were collected from brown and black colonieson carved dragon column pedestal made of white marble. (F) shows sample QX7, which was taken from green biofilms covering the surface of a stone building built in Qing dynasty.
(PDF)

S2 Fig SEM-EDS analysis of samples QX4 and QX7. The composition of the white marble in Qingxing palace is aluminum oxide and silicon dioxide.
(PDF)

S3 Fig SEM-EDS analysis of samples LY2 and LY3. The stone mainly contains sulfur, silicon, oxygen and calcium, and small amounts of carbon and aluminum. These results indicate that the stone consists of a series of compounds including calcium silicate, aluminum oxide, silicon dioxide and sulfide.
(PDF)

S4 Fig Rarefaction analysis for the observed number of (A) bacterial OTUs and (B) fungal OTUs.
(PDF)

S5 Fig **Aheatmap of β diversity for (A) bacterial communities and (B) fungal communities.** Weighted UniFrac UPGMA tree based on bacterial and archaeal V4 16S rRNA gene sequences and fungal V2 ITS gene sequences obtained from six stone monuments located in Hangzhou city, Zhejiang province. The heat map shows the relative abundance and diversity distance within each sample. The abundance data were normalized by range-scaling each class 0–1.
(PDF)

S6 Fig Environmental parameters at the six sites showing monthly average (A) temperature, (B) light intensity, (C) concentrations of NO_2_ and SO_2_, and (D) air humidity and sample humidity.
(PDF)

S7 Fig ITS gene sequencing reveals the relationship and membership of the stone monuments colonies. Weighted UniFrac UPGMA tree based on fungal V4 ITS gene sequences obtained from six stone monuments located in Hangzhou city, Zhejiang province.
(PDF)

S1 Table Collation of alpha diversity results from bacteria. (DOC)

S2 Table Collation of alpha diversity results from fungi. (DOC)
