# Supplementary figures and images for: Distribution and Diversity of Bacteria and Fungi Colonization in Stone Monuments Analyzed by High-Throughput Sequencing
Source: PLoS One. 2016 Sep 22;11(9):e0163287. doi: 10.1371/journal.pone.0163287 (PMC5033376; doi:10.1371/journal.pone.0163287)

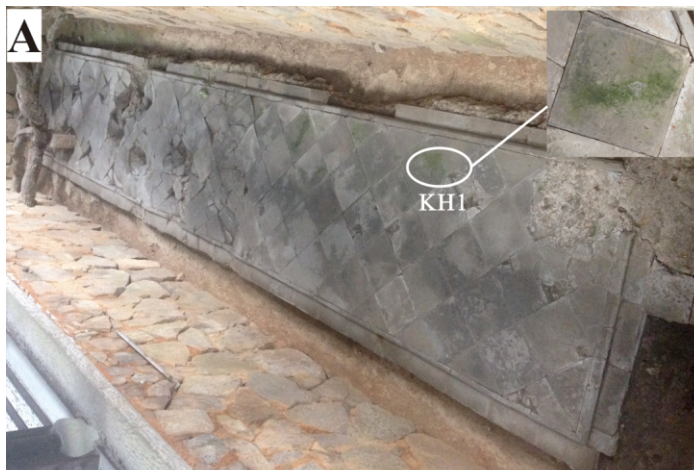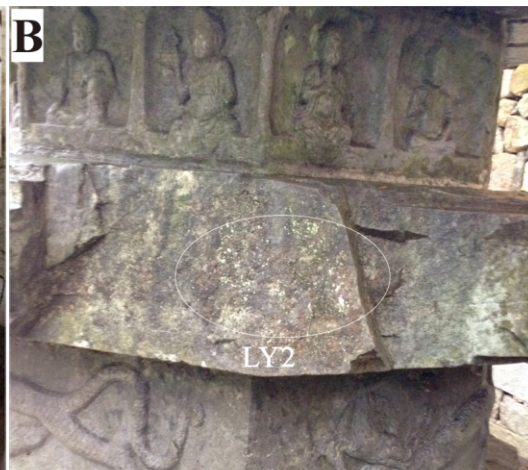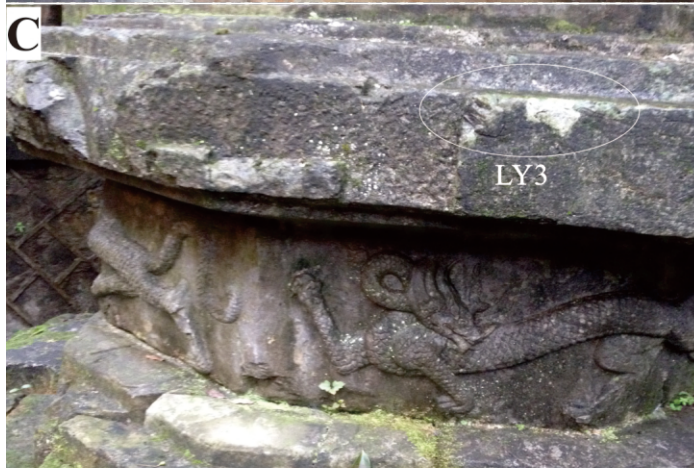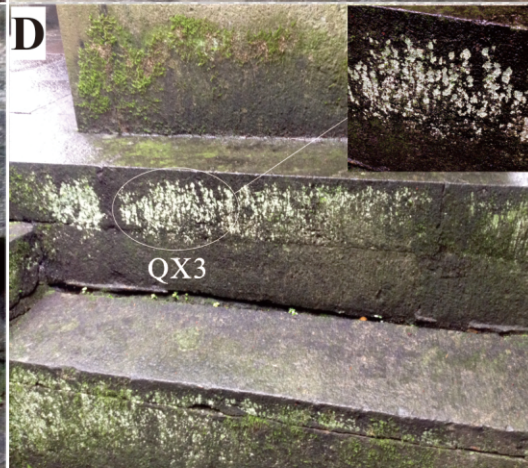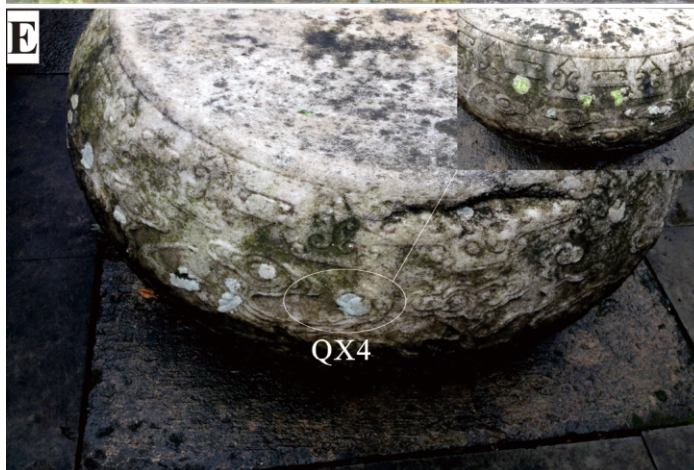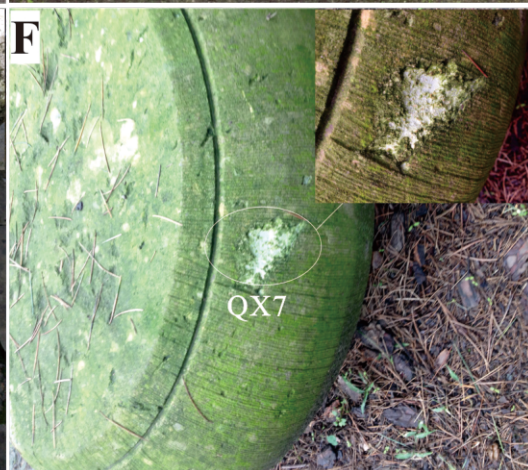

Supplement: S1 Fig — (A) shows sample KH1, which was collected from the green biofilms on the surface of the ancient bricks located in Kaihua temple. (B) and (C) show samples LY2 and LY3, which were collected from green and white colonies located on the Buddhist stone pillars of Lingyin temple. (D) and (E) show samples QX3 and QX4, which were collected from brown and black colonieson carved dragon column pedestal made of white marble. (F) shows sample QX7, which was taken from green biofilms covering the surface of a stone building built in Qing dynasty. (PDF) [file pone.0163287.s001.pdf]

# SEM

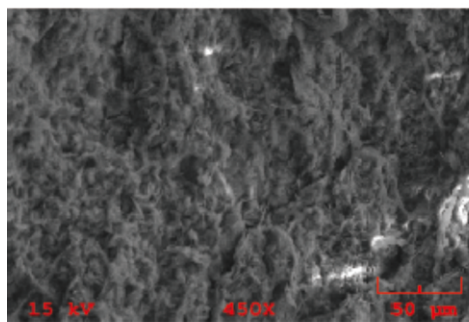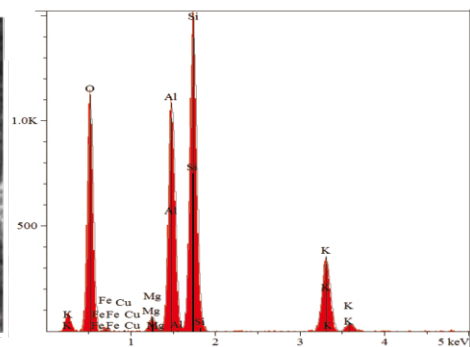

Al

O

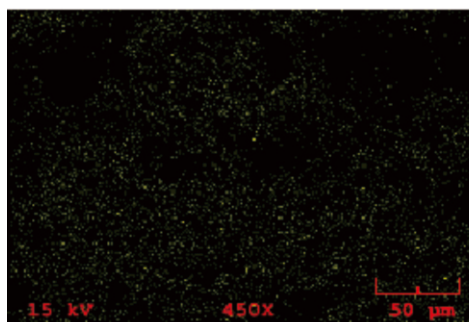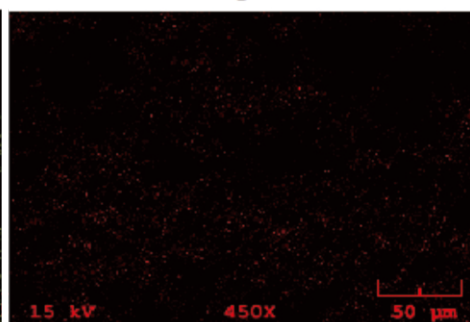

Fe

Si

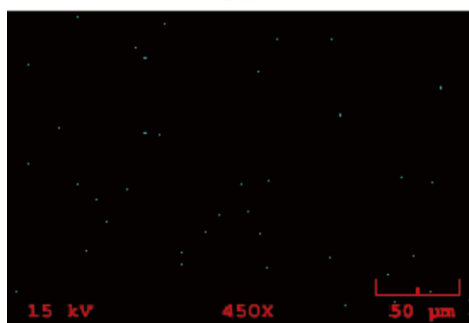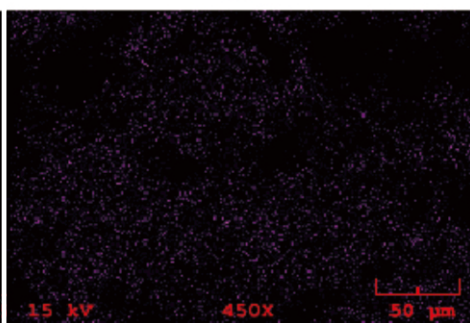

Supplement: S2 Fig — The composition of the white marble in Qingxing palace is aluminum oxide and silicon dioxide. (PDF) [file pone.0163287.s002.pdf]

# SEM

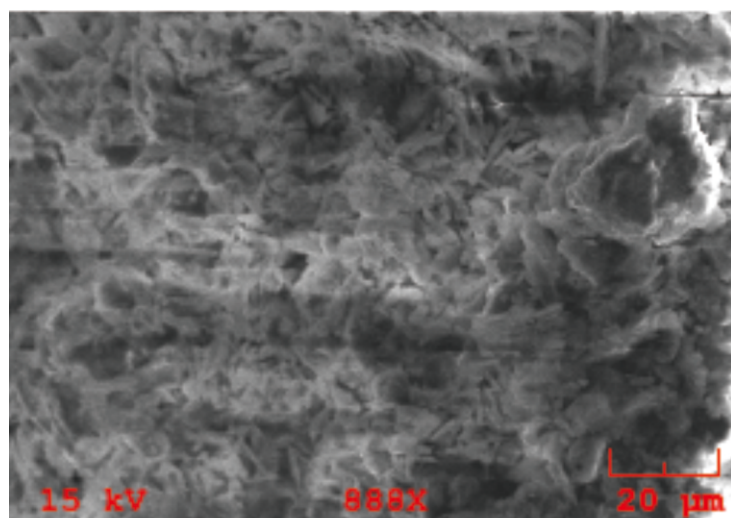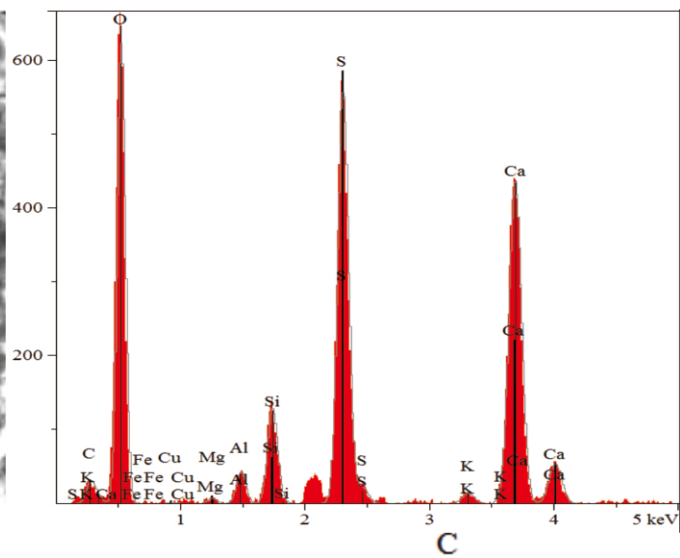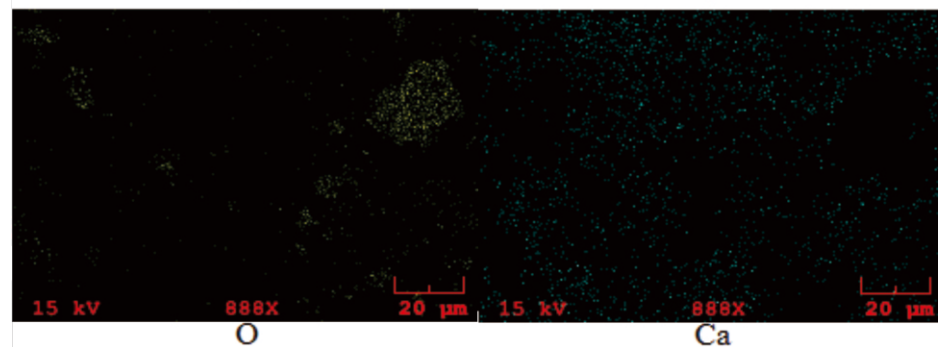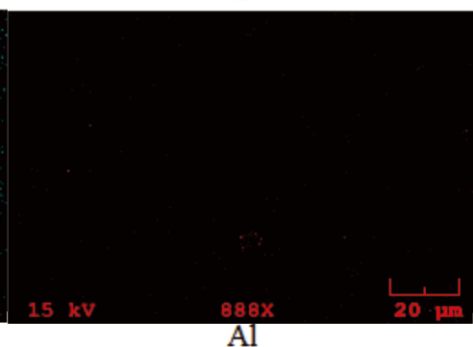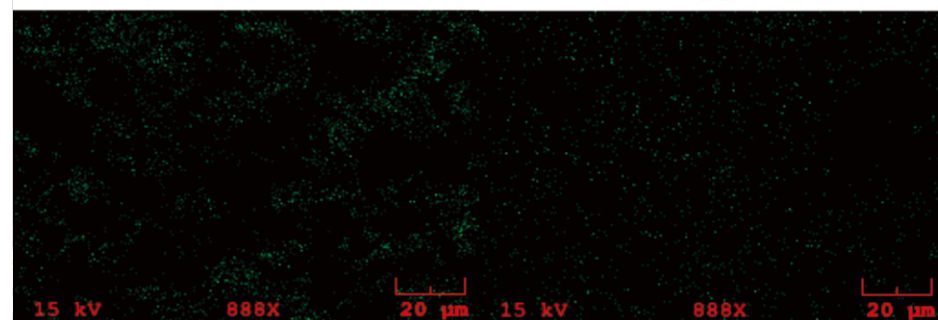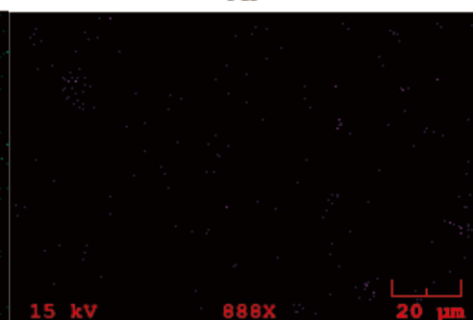

Supplement: S3 Fig — The stone mainly contains sulfur, silicon, oxygen and calcium, and small amounts of carbon and aluminum. These results indicate that the stone consists of a series of compounds including calcium silicate, aluminum oxide, silicon dioxide and sulfide. (PDF) [file pone.0163287.s003.pdf]

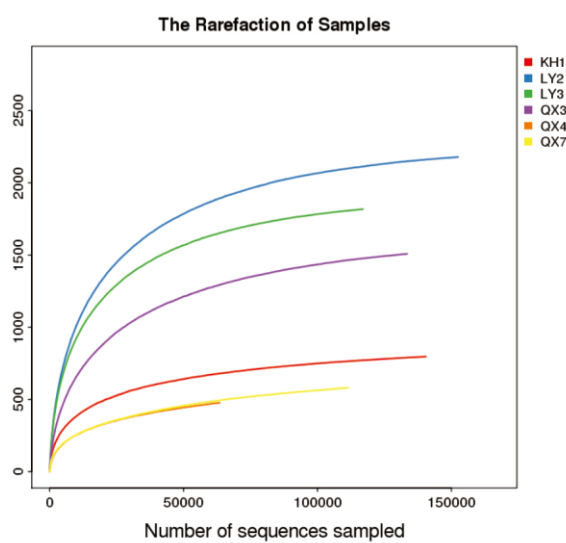

A

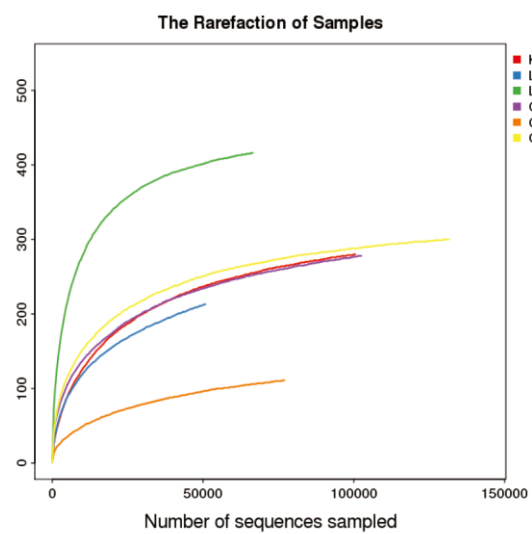

B

Supplement: S4 Fig — Rarefaction analysis for the observed number of (A) bacterial OTUs and (B) fungal OTUs. (PDF) [file pone.0163287.s004.pdf]

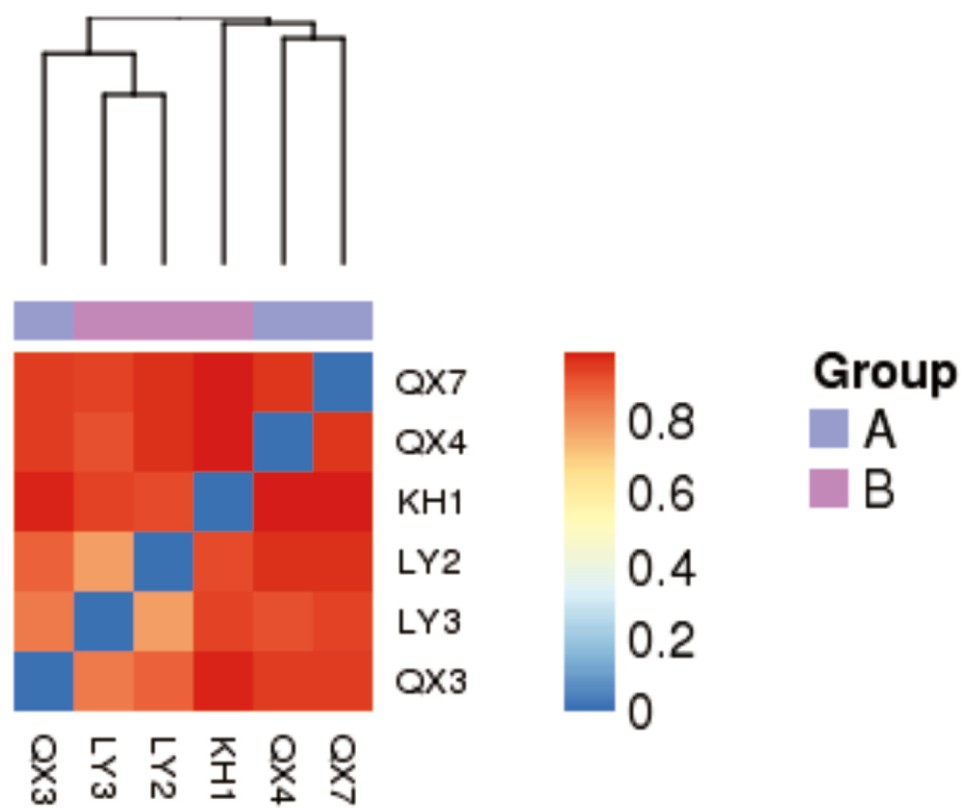

(A)

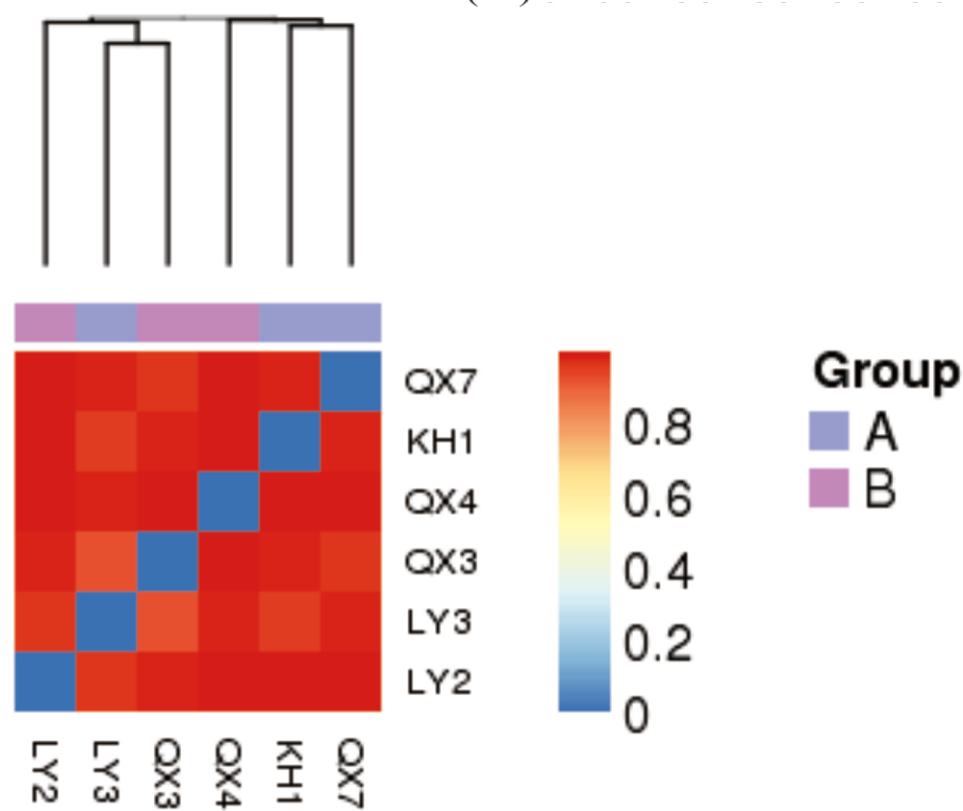

(B)

Supplement: S5 Fig — Aheatmap of β diversity for (A) bacterial communities and (B) fungal communities. Weighted UniFrac UPGMA tree based on bacterial and archaeal V4 16S rRNA gene sequences and fungal V2 ITS gene sequences obtained from six stone monuments located in Hangzhou city, Zhejiang province. The heat map shows the relative abundance and diversity distance within each sample. The abundance data were normalized by range-scaling each class 0–1. (PDF) [file pone.0163287.s005.pdf]

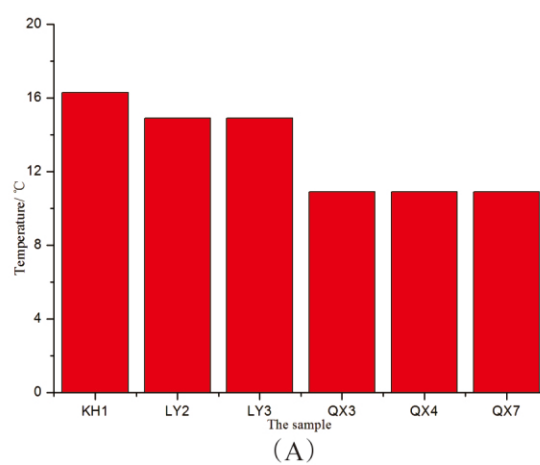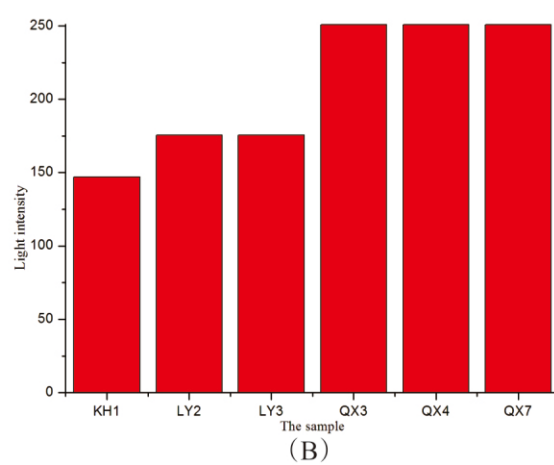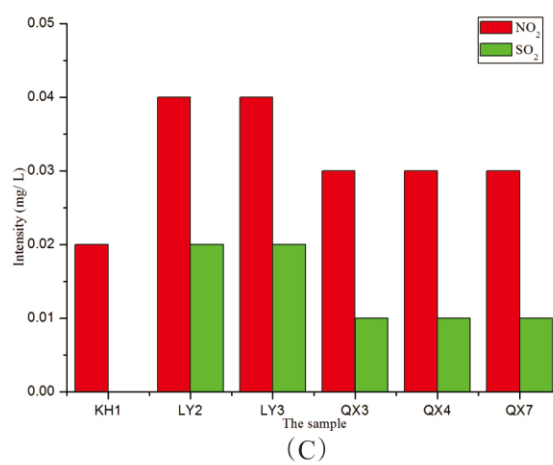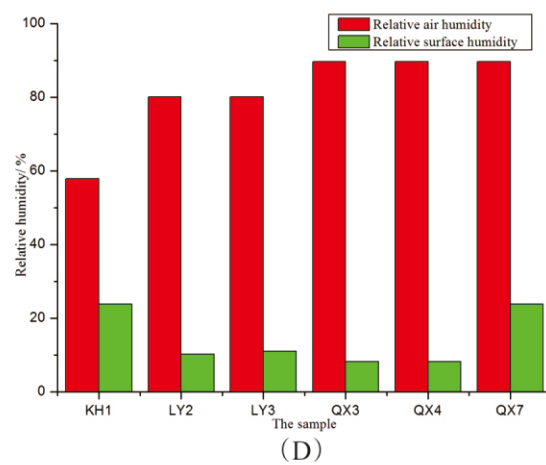

Supplement: S6 Fig — Environmental parameters at the six sites showing monthly average (A) temperature, (B) light intensity, (C) concentrations of NO2 and SO2, and (D) air humidity and sample humidity. (PDF) [file pone.0163287.s006.pdf]

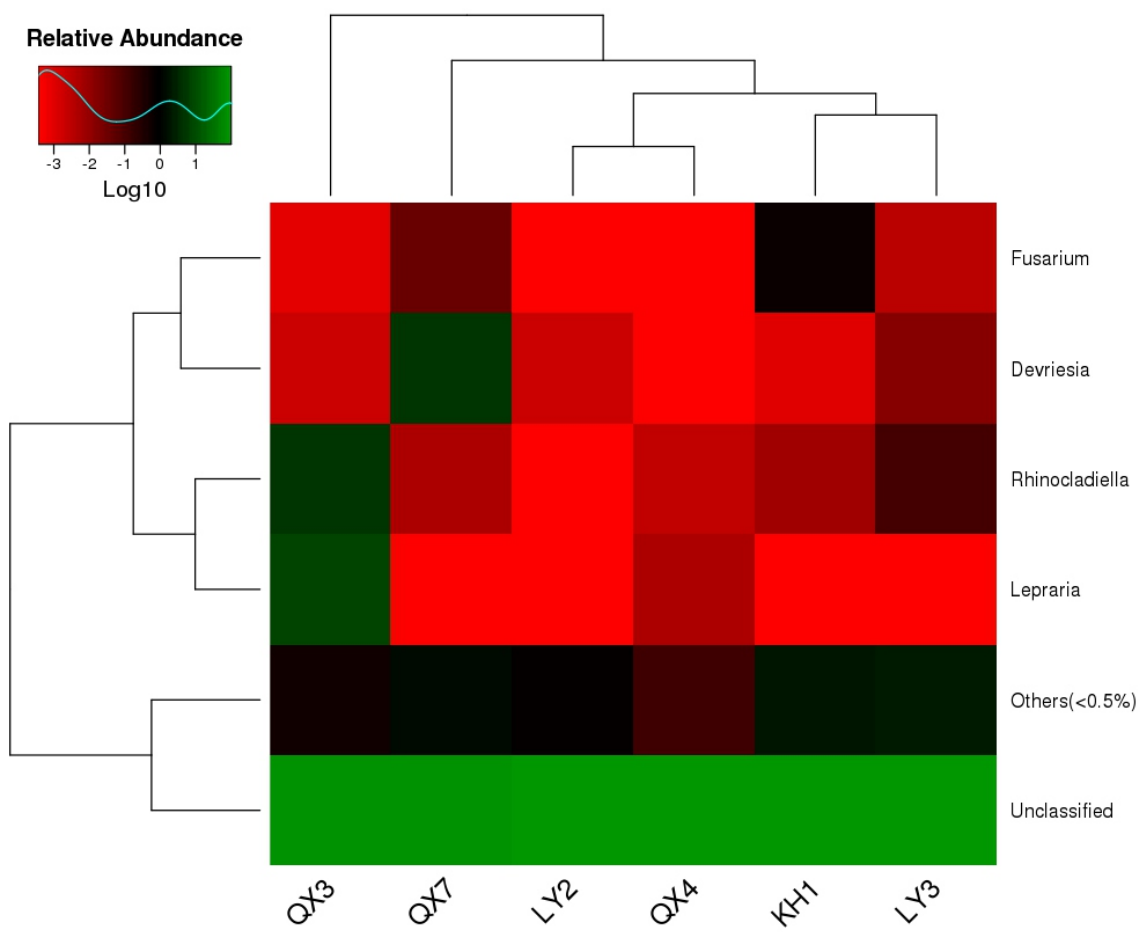

Supplement: S7 Fig — Weighted UniFrac UPGMA tree based on fungal V4 ITS gene sequences obtained from six stone monuments located in Hangzhou city, Zhejiang province. (PDF) [file pone.0163287.s007.pdf]
