# Supplementary material for: Distribution and Diversity of Bacteria and Fungi Colonization in Stone Monuments Analyzed by High-Throughput Sequencing
Source: PLoS One. 2016 Sep 22;11(9):e0163287. doi: 10.1371/journal.pone.0163287 (PMC5033376; doi:10.1371/journal.pone.0163287)
Supplement: S1 Table — (DOC) [file pone.0163287.s008.doc]

**S1 Table** Collation of alpha diversity results from bacteria.

|  | Observed species | ACE | Chao | Shannon | Simpson |
| --- | --- | --- | --- | --- | --- |
| KH1-16S | 797 | 891.11 | 896.75 | 3.672269 | 0.066974 |
| LY2-16S | 2179 | 2268.08 | 2241.79 | 4.451088 | 0.078649 |
| LY3-16S | 1818 | 1917.23 | 1899.12 | 4.813508 | 0.040555 |
| QX3-16S | 1508 | 1640.83 | 1620.00 | 3.053443 | 0.321422 |
| QX4-16S | 477 | 621.31 | 636.85 | 2.223191 | 0.355860 |
| QX7-16S | 581 | 733.42 | 719.43 | 2.546926 | 0.303958 |
