# Supplementary material for: Distribution and Diversity of Bacteria and Fungi Colonization in Stone Monuments Analyzed by High-Throughput Sequencing
Source: PLoS One. 2016 Sep 22;11(9):e0163287. doi: 10.1371/journal.pone.0163287 (PMC5033376; doi:10.1371/journal.pone.0163287)
Supplement: S2 Table — (DOC) [file pone.0163287.s009.doc]

**S2 Table** Collation of alpha diversity results from fungi.

|  | Observed species | ACE | Chao | Shannon | Simpson |
| --- | --- | --- | --- | --- | --- |
| KH1-ITS | 280 | 317.73 | 318.5 | 1.346344 | 0.434495 |
| LY2- ITS | 213 | 283.64 | 289.6 | 1.257874 | 0.409536 |
| LY3- ITS | 416 | 435.39 | 436.5 | 3.354797 | 0.074251 |
| QX3- ITS | 278 | 325.27 | 343.3 | 2.346263 | 0.232444 |
| QX4- ITS | 111 | 145.93 | 146.0 | 1.239379 | 0.375909 |
| QX7- ITS | 300 | 325.35 | 329.7 | 2.557888 | 0.166081 |
